# Supplementary material for: Resolving the full spectrum of human genome variation using Linked-Reads
Source: Genome Res. 2019 Apr;29(4):635–45. doi: 10.1101/gr.234443.118 (PMC6442396; doi:10.1101/gr.234443.118)
Supplement: Supplemental Material [file supp_gr.234443.118_Supplemental_longranger-2.2.zip › longranger-2.2.tar.bz2!/lr2.2-public/lib/stan/cmdstan/stan/doc/api/html/values_8hpp_source.html]

Stan: src/stan/common/recorder/values.hpp Source File


|  |  |
| --- | --- |
|  | Stan  2.5.0  probability, sampling & optimization |


- Main Page
- Related Pages
- Namespaces
- Classes
- Files

- File List
- File Members

All Classes Namespaces Files Functions Variables Typedefs Enumerations Enumerator Friends Macros Pages

- src
- stan
- common
- recorder

values.hpp

Go to the documentation of this file.

1 #ifndef STAN\_\_COMMON\_\_RECORDER\_\_VALUES\_HPP

2 #define STAN\_\_COMMON\_\_RECORDER\_\_VALUES\_HPP

3

4 #include <ostream>

5 #include <string>

6 #include <stdexcept>

7 #include <vector>

8

9 namespace stan {

10  namespace common {

11  namespace recorder {

12

13  template <class InternalVector>

14  class values {

15  private:

16  size\_t m\_;

17  size\_t N\_;

18  size\_t M\_;

19  std::vector<InternalVector> x\_;

20

21  public:

22

23  values(const size\_t N,

24  const size\_t M)

25  : m\_(0), N\_(N), M\_(M) {

26  x\_.reserve(N\_);

27  for (size\_t n = 0; n < N\_; n++)

28  x\_.push\_back(InternalVector(M\_));

29  }

30

31  values(const std::vector<InternalVector>& x)

32  : m\_(0), N\_(x.size()), M\_(0),

33  x\_(x) {

34  if (N\_ > 0)

35  M\_ = x\_[0].size();

36  }

37

38  void operator()(const std::vector<std::string>& x) { }

39

40  template <class T>

41  void operator()(const std::vector<T>& x) {

42  if (N\_ != x.size())

43  throw std::length\_error("vector provided does not match the parameter length");

44  if (m\_ == M\_)

45  throw std::out\_of\_range("");

46  for (size\_t n = 0; n < N\_; n++)

47  x\_[n][m\_] = x[n];

48  m\_++;

49  }

50

51  void operator()(const std::string x) { }

52

53  void operator()() { }

54

55  bool is\_recording() const {

56  if (m\_ < M\_)

57  return true;

58  return false;

59  }

60

61  const std::vector<InternalVector>& x() const {

62  return x\_;

63  }

64  };

65

66  }

67  }

68 }

69

70 #endif

stan::common::recorder::values::operator()

void operator()()

**Definition:** values.hpp:53

stan::common::recorder::values::values

values(const size\_t N, const size\_t M)

**Definition:** values.hpp:23

stan::common::recorder::values::operator()

void operator()(const std::string x)

**Definition:** values.hpp:51

stan::common::recorder::values::is\_recording

bool is\_recording() const

**Definition:** values.hpp:55

stan::common::recorder::values

**Definition:** values.hpp:14

stan::common::recorder::values::operator()

void operator()(const std::vector< T > &x)

**Definition:** values.hpp:41

stan::common::recorder::values::x

const std::vector< InternalVector > & x() const

**Definition:** values.hpp:61

stan::common::recorder::values::values

values(const std::vector< InternalVector > &x)

**Definition:** values.hpp:31

stan::math::size

int size(const std::vector< T > &x)

**Definition:** size.hpp:11

stan::common::recorder::values::operator()

void operator()(const std::vector< std::string > &x)

**Definition:** values.hpp:38


---

[ Stan Home Page ]

*© 2011–2014,
Stan Development Team.*
